# The conceptually equivalent Dutch version of the Western Ontario Rotator Cuff Index (WORC)©

**DOI:** 10.1186/1471-2474-14-362

**Published:** 2013-12-21

**Authors:** Ronald N Wessel, Nienke Wolterbeek, Anouk JM Fermont, Henk van Mameren, Heleen Sonneveld, Sharon Griffin, Rob A de Bie

**Affiliations:** 1 Department of Orthopaedic Surgery, St. Antonius Ziekenhuis, PO Box 2500, Nieuwegein 3430, EM, The Netherlands; 2 Department of Epidemiology, Caphri research school, Maastricht University, PO Box 616, Maastricht 6200, MD, The Netherlands; 3 Department of Orthopaedic Surgery, Meander Medisch Centrum, PO Box 1502, Amersfoort 3800, BM, The Netherlands; 4 Fowler Kennedy Sport Medicine Clinic, University of Western Ontario, London N6A 3 K7, Canada; 5 Department of Epidemiology, Caphri research school, Maastricht University, PO Box 616, Maastricht 6200, MD, The Netherlands

**Keywords:** WORC, Quality of life questionnaires, Translation process, Validation, Rotator cuff repair

## Abstract

**Background:**

The WORC is a quality of life questionnaire designed for patients with disorders of the rotator cuff, originally developed in English. The purpose of this study was to cross-culturally adapt the WORC for use in the Dutch population and to evaluate reliability, agreement and floor and ceiling effects of this Dutch version in a population of patients with rotator cuff disease.

**Methods:**

Reliability was tested by measuring the Cronbach’s alpha for internal consistency and intraclass correlation coefficients (ICC) for test-retest reliability. Agreement was measured using the Standard Error of Measurement (SEM_agreement_); and the smallest detectable change (SDC) was calculated based on the SEM. Pearson Correlations Coefficients were used to comparing the WORC with the RAND-36, the Constant Score and 11-point shoulder hindrance scale.

**Results:**

Fifty-seven patients entered into this study of whom 50 were available for test-retest validation. The internal consistency of the Dutch WORC tested by Cronbach’s alpha was 0.95 for the total questionnaire. The ICC for the WORC is 0.91 with a 95% confidence interval of 0.85-0.95. Standard Error of Measurement was 6.0 points with a Smallest Detectable Change of 16.7 points on a 0-100 scale. Pearson Correlations Coefficients showed a significant positive correlation between the Dutch WORC and Constant Score (r = 0.60) and a strong reversed correlation with the shoulder hindrance scale (r = -0.75).

**Conclusion:**

The Dutch WORC seems to be a reliable health-related quality of life questionnaire for patients with rotator cuff disorders.

**Trial registration:**

NCT01532492.

## Background

Arthroscopic repair of a rotator cuff tear is a procedure that has gained increased interest. Results are reported to be good to excellent in more than 90% of patients [1-9]. There is however no consensus on how to measure success after this procedure. Results can be measured by patient satisfaction, cuff continuity or clinical scores. Examples of widely used outcome measures in rotator cuff repair studies are the Constant Score and the UCLA Shoulder Score. Dutch quality of life measurement tools specifically suitable to investigate patients with diseases of the rotator cuff are lacking in the literature.

Disease specific quality of life measurement tools are essential for evaluating orthopedic healthcare, since the goal of orthopedic surgery is to improve quality of life rather than to prolong a patient’s life [10]. If the questionnaires are self-administered then they are less susceptible to evaluator bias. It is important to measure results from a patient’s point of view rather than from a practitioner’s perspective.

Multiple questionnaires concerning shoulder pathology have been developed [11-19]. The Western Ontario Rotator Cuff index (WORC) and the Rotator Cuff Quality-of-Life Measure (RC-QOL) are disease specific quality of life measurement tools for patients with rotator cuff disease. We chose to translate and validate the WORC, as it was developed for people with rotator cuff disease with well described methodology that included item generation and reduction, scaling and weighting, pretesting, reliability and validation testing [14]. Furthermore, a common international interpretation and analysis of results is only possible if data comes from the same instrument [20]. The WORC has been translated into a number of languages [21-25], which makes this measurement tool appropriate for international comparison of results.

The purpose of this study was to cross-culturally adapt the WORC for use in the Dutch population and to evaluate reliability, agreement and floor and ceiling effects of this Dutch version in a population of patients with rotator cuff disease.

## Methods

In this prospective study the WORC index was translated and adapted into the Dutch language. After the translation process was completed, the questionnaire was evaluated in a population of patients with rotator cuff disease. It was compared to the RAND-36 [26] and the Constant Score.

### WORC

The WORC is designed for patients with disorders of the rotator cuff [10]. It is a disease-specific Health Related Quality of Life (HRQL) questionnaire that has 21 items representing 5 domains, each with a visual analogue scale–type response option. The 5 domains are (1) physical symptoms, (2) sports and recreation, (3) work, (4) social function, and (5) emotions. Each item is scored on a 100-mm scale (ranging from 0 best to 100 worst). The most symptomatic total score is 2100, and the best or asymptomatic total score is 0. To present this in a more clinically meaningful format, the score is reported as a percentage by subtracting the total from 2100, dividing by 2100, and multiplying by 100. Total final WORC scores can, therefore, vary from 0%, the lowest functional status level, to 100%, the highest functional status level. In this article both the WORC total and domain scores are expressed as percentages (0-100%).

### RAND-36

The RAND 36-Item Health Survey 1.0 (distributed by RAND, [26]) includes the same items as those found in the SF-36, but with a scoring algorithm that is somewhat different from that of the SF-36 [27]. The RAND-36 is a widely used HRQL survey instrument. It assesses eight health concepts with multi-item scales (35 items): physical functioning (10 items), role limitations caused by physical health problems (4 items), role limitations caused by emotional problems (3 items), social functioning (2 items), emotional well being (5 items), energy/fatigue (4 items), pain (2 items) and general health perceptions (5 items). An additional single item assesses change in perceived health during the last 12 months [26,28].

### Constant score

The Constant Score has become the most widely used shoulder evaluation instrument in Europe. It is a 100-point scoring system and combines physical examination tests with subjective evaluations by the patients. The subjective assessments of pain and activities of daily living are allocated 15 and 20 points, respectively. A maximum of 40 points is assigned for active range of motion, and 25 points for quantitative measurement of abduction strength [14,29,30].

### Translation

A simple, literal translation of a quality of life questionnaire does not suffice, because of linguistic and cultural differences between countries. It is important that a translated questionnaire is not only a linguistic equivalent of its original but also comprehensible equal. Therefore, we created a tool that is conceptually equivalent to the original English questionnaire following the steps of the MAPI method [31].

1. “Forward” translation by two independent translators

One translation was performed by a professional translator with a medical background. The second translation was performed by an orthopedic surgeon together with a native English speaker who lived in the Netherlands for more than 20 years.

2. Reconciliation meeting between the two “forward” translators and the local project manager

The two forward translations were merged into one forward translation by a committee consisting of two of the authors and the forward translators. Although not described in the translation guidelines we tested this first draft version on 10 patients with rotator cuff disease including bursitis, impingement, tendonitis and rupture, to get an impression of the understandability. It was found that patients were confused about the slash as a mark on the 100 mm horizontal lines. We therefore allowed patients to mark the lines with a cross “X”.

3. “Backward” translation by an independent translator

The translation mentioned at step 2 was translated back into English by a Dutch orthopedic surgeon.

4. Comparison of the source questionnaire with the “backward” translation

**Figure 1:**
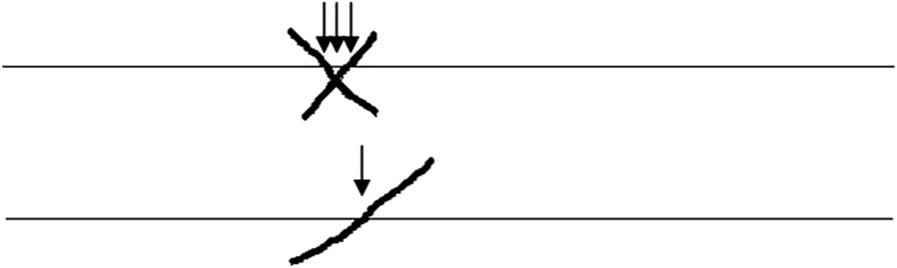
**Three different values can be measured by marking with a cross “X”.** Marking with a slash ‘/’ only one value can be measured.

This back-translation was sent to an author (SG) of the original questionnaire for comments. In Table 1 the phrases are outlined which have not been translated literally. There were no major differences between the original test and the back-translation, except for the adaptation of the mark (cross “X” instead of a slash “/”, see 2). At first, the back-translation was not approved because of this change. Marking with a cross “X” requires the exact measurement on the 100 mm line, since three possibilities exist (Figure 1). By changing it back to the “slash” we also bypassed the problem of measuring the position of the cross-mark on the line.

**Table 1:** List questions and explanations, which have not been translated literally

	**English**	**Dutch**	**Explanation**
Question 9	How much difficulty do you have with someone or something coming in contact with your affected shoulder?	Hoe bang bent u dat iets of iemand tegen uw geblesseerde schouder stoot?	How much difficulty is translates as How afraid are you
Question 12	How much difficulty do you experience working above your head?	Hoeveel last ervaart u bij het verrichten van werkzaamheden boven schouderhoogte?	Above your head is translated as above shoulder level
Explanation 16	Refers to anything that you would do to your hair such as combing, brushing or washing that requires you to reach up with your problematic arm.	Heeft betrekking op alles wat er bij haarverzorging komt kijken, zoals kammen, borstelen of wassen, waarbij u uw aangedane arm moet optillen. Indien u schouderklachten heeft, maar daar geen last van heeft bij de verzorging van uw haar (bijvoorbeeld omdat dat prima gaat met uw andere arm of dat u kalend bent), zet u een streepje links op de lijn.	The explanation was expanded by: If you have shoulder complaints, but they do not bother you when styling your hair (because it goes fine with the other arm) you put a slash left on the line. If you do not experience difficulty styling your hair because your are balding, indicate your difficulty to wash the top of your head
Question 17	How much difficulty do you have “roughhousing or horsing around” with family or friends?	Hoeveel last hebt u met ‘stoeien en ravotten’ met familie/vrienden vanwege uw schouder?	The explanation was adjusted by adding ‘*e.g. grandchildren*’

5. Review by clinicians working in the medical field

The WORC was then reviewed by two different orthopedic surgeons both with extensive experience with the English language, a physical therapist and a patient counselor. Issues such as how to define ‘working above your head’ and ‘at or below shoulder level’ were discussed. No major changes were necessary.

6. Cognitive debriefing

Clarity, understandability and acceptability of the WORC were tested on 15 people both with and without shoulder complaints, followed by face to face interviews. The results were discussed with the members of the committee. There were no missing items. Bald people had difficulty answering question 16 (How much difficulty have you experienced with styling your hair because of your shoulder?). Therefore, the explanation was adjusted by adding ‘If you have shoulder complaints, but they do not bother you when styling your hair (because it goes fine with the other arm) you put a slash left on the line. If you do not experience difficulty styling your hair because your are bald, indicate your difficulty to wash the top of your head’. We found that elderly people had trouble imagining the activity referred to in question 17 (How much difficulty do you have “roughhousing and horsing around” with family or friends?). The explanation was adjusted by adding ‘e.g. grandchildren’. Furthermore people had difficulty answering questions that did not pertain to them, e.g. question 10 (How much difficulty do you experience doing push-ups or other strenuous shoulder exercises because of your shoulder?) in elderly. Another example is question 21 (How worried or concerned are you about the effect of your shoulder on your occupation?) in people who have been retired. After consulting with the author a consensus was reached. In the introduction of the questionnaire it is pointed out that patients who did not experience an item should “imagine” how that item would affect them. The direction of the mark was also discussed since it may feel odd for left-handed persons to put a slash ”/” instead of a backslash “\” on the line . The author stated that the direction of the mark does not really matter as long as it crosses the line.

7. Report

After reporting the process and the final translation of the WORC (Additional file 1) we received approval from the author to use this questionnaire for the Dutch population.

### Patients

Patients above the age of 18 with at least 3 months duration of disorders of the rotator cuff (DRC), including both rotator cuff lesion and non-ruptured disorders of the rotator cuff, were prospectively included in this study. The patients were recruited from the outpatient clinic of the St Antonius Hospital in Utrecht, the Netherlands and had to have two or more of the following signs present: impingement (Neer’s impingement sign or Hawkins-Kennedy impingement sign), painful arc sign, positive Jobe test (supraspinatus), positive infraspinatus test (resistance test with external rotation at the side and in 90 degrees of abduction), positive lift-off/belly press test (resistance test of subscapularis) [32], positive drop-arm test [33], or positive Neer impingement test [32] (subacromial injection with lidocaine). The lesions were confirmed by Magnetic Resonance Imaging (MRI) or arthroscopy. Calcific tendonitis was confirmed on X-ray [34]. Exclusion criteria was previous operations and co-existing pathology such as osteoarthritis, frozen shoulder or instability of the affected shoulder. Patients who had difficulty understanding the Dutch language were also excluded.

### Visits

The visits for the validation study were scheduled with an independent physical therapist with a two week interval between visits (minimum of one week). During these two visits (T0 and T1), patients filled out the WORC, RAND-36 (general health) and the Constant Score in a separate room with the independent physical therapist in attendance. They were also asked to rate their shoulder hindrance on an 11-point scale from 0 (no hindrance) to 10 points (extreme hindrance). The one to two week test-retest interval was chosen because it was unlikely that the patient’s condition would substantially change, but the time span was large enough for the patients to forget their initial responses to the questions. In order to detect a possible substantial change in the patient’s condition, the shoulder hindrance scale was used. When the difference between T0 and T1 on this scale was more than 2 points, the patient was excluded from the test-retest analysis, regardless if this was caused by natural variation or by an actual change in condition. The protocol was approved by the institutional review board of the St. Antonius Hospital and all patients gave informed consent.

### Statistics

Descriptive statistics were compiled for demographic and clinical characteristics of the study population. Based on the general recommendations for comparing measurement properties, at least 50 patients need to be included [35]. Pearson Correlations Coefficients (r) were used to compare the Dutch WORC with a general quality of life questionnaire (RAND-36), a commonly used clinical shoulder score (Constant Score) and an 11-point shoulder hindrance scale. For this analysis, the data from the first administration (T0) of the questionnaires was used. Floor and ceiling effects were considered to be present if more than 15% of the patients achieved respectively the lowest or highest possible score [36]. If floor or ceiling effects are present, patients with the lowest or highest possible score cannot be distinguished from each other, indicating limited content validity [35]. All data was analyzed with SPSS statistical software (SPSS, Inc., version 19). For statistical analyses, the level of significance was set at 5%.

### Agreement and reliability

Cronbach’s alpha coefficients were calculated for internal consistency of the WORC total score and the five domains [37]. An alpha <0.6 indicates a poor internal consistency, 0.7-0.8 acceptable, 0.8-0.9 good and >0.9 excellent internal consistency. High internal consistency indicates a strong correlation between the items, which supports summarizing the items [35]. Intraclass correlation coefficients (ICC) were calculated for test-retest reliability of the WORC total score and the five domains (two-way random effects model, single measurements and absolute agreement) [38]. In general, 0.70 is recommended as a minimum standard for test-retest reliability; a correlation less than 0.5 is described as weak, whereas a correlation greater than 0.8 is described as strong [39]. The 95% confidence interval (CI) for the ICC, the range of values contained with the 95% confidence (the ‘true’ correlation coefficient), were also calculated. Agreement was measured using the Standard Error of Measurement (SEM) calculated as SEM_agreement_[40]. The larger the SEM, the lower the precision of the instrument. The smallest detectable change (SDC), based on the measurement error, was defined as 1.96 * √2 * SEM [40].

## Results

### Validation

Fifty-seven patients were included in this study. Demographics and clinical characteristics are presented in Tables 2 and 3. The average age was 54.2 years (range 26-86 years); 27 patients (47%) were female and thirty patients (53%) suffered from rotator cuff tear. The right shoulder was involved in 28 patients (49%) and the dominant shoulder was involved in 53 patients (93%).

**Table 2:** Demographics of study population (N = 57)

**Female/male**	**27/30**
Diagnosis	
Rotator cuff tear	30
Calcific tendonitis	12
Impingement/tendinosis/tendonitis	15
Operated side (Right/Left)	28/29
Dominant side (Right/Left)	53/4

**Table 3:** Clinical characteristics of study population at the first administration of the questionnaires (N = 57)

	**Mean (±SD)**	**Range**
Age (years)	54.2 (± 10.8)	24-75
Interval T0-T1 (days)	8.6 (± 3.0)	7-14
WORC index total	44.2 (± 21.2)	6-96
WORC-physical symptoms	53.9 (± 20.6)	9-96
WORC-sports and recreation	38.8 (± 24.1)	1-99
WORC-work	33.8 (± 23.8)	0-99
WORC-lifestyle	39.7 (± 24.8)	1-93
WORC-emotions	51.6 (± 29.1)	0-99
Constant score	36.0 (± 20.6)	2-85
Shoulder hindrance	7.2 (± 2.0)	2-10
RAND-36		
Physical functioning	71.8 (± 14.4)	25-100
Social functioning	67.1 (± 26.2)	0-100
Physical role	23.3 (± 33.4)	0-100
Emotional role	63.2 (± 44.4)	0-100
Mental health	75.9 (± 18.0)	12-100
Vitality	61.4 (± 20.6)	15-100
Bodily pain	42.0 (± 20.5)	0-90
General health	65.9 (± 20.2)	15-95
Reported health	36.8 (± 22.7)	0-100
Physical health (summary score)	52.9 (± 14.7)	28-87
Mental health (summary score)	66.7 (± 19.4)	13-95

WORC: Western Ontario Rotator Cuff Index.

Shoulder hindrance: 11-point scale from 0 (no hindrance) to 10 points (extreme hindrance).

Domain scores WORC presented from 0 (worst possible) to 100 (best possible).

There was a significant positive correlation between the Dutch WORC and Constant Score (r = 0.60). The correlations were stronger with the physical health summary scale than with the mental health summary scale of the RAND-36. There was also a strong reversed correlation demonstrated between experienced shoulder hindrance and the WORC (r = -0.75) (Table 4).

**Table 4:** Pearson correlations coefficient between WORC, constant score, RAND-36 and Shoulder hindrance (N = 57)

	**Correlation coefficients (r)**	**P**
	**WORC total**	
Constant Score	0.60	<0.001*
RAND-36		
Physical health (summary score)	0.66	<0.001*
Mental health (summary score)	0.46	<0.001*
Shoulder hindrance	-0.75	<0.001*

WORC: Western Ontario Rotator Cuff Index.

*Correlation is significant at the 0.05 level (2-tailed).

There were no ceiling effects for the different domains of the WORC. Two patients (3.5%) had a minimal score (worst possible), in the ‘Emotions’ and ‘Work’ domains. There were no floor or ceiling effects for the total WORC. No floor effects on the shoulder hindrance scale were seen, however, 5 patients (8.8%) had the maximum score (extreme hindrance). No floor or ceiling effects were present for the Constant Score or for the summary scores ‘Physical health’ and ‘Mental health’ of the RAND-36. Since the questionnaires were filled out in the present of an independent physical therapist, there was no missing data.

### Agreement and reliability

Internal consistency of the Dutch WORC tested by Cronbach’s Alpha was 0.95 for the total questionnaire. The results for the five domains are shown in Table 5. Five patients did not have a T1 administration. Two patients with a rotator cuff tear were excluded from the test-retest analysis because they showed a difference of more than two points (3 and 4 points) on the shoulder hindrance scale. Therefore 50 patients were included in the test-retest analysis. The ICC for the WORC was 0.91 with a 95% confidence interval of 0.85-0.95. The ICC of the five domains ranged between 0.79 and 0.89. SEM and SDC for the WORC total score was respectively 6.0 and 16.7 points on the 0-100 scale. For the different domain the SEM varied between 8.4 and 12.9 points and the SDC between 23.3 and 35.9 points (Table 5).

**Table 5:** Cronbach’s Alpha, Intraclass Correlation Coefficients (ICC), Standard error of Measurement (SEM) and Smallest Detectable Change (SDC) of the Dutch version of the WORC (0-100)

	**Cronbach’s Alpha**	**ICC (95% CI)**	**SEM**	**SDC**
WORC score total	0.95	0.91 (0.85-0.95)	6.0	16.7
WORC-physical symptoms	0.82	0.79 (0.65-0.88)	10.0	27.6
WORC-sports and recreation	0.79	0.88 (0.79-0.93)	8.4	23.4
WORC-work	0.85	0.87 (0.78-0.92)	8.4	23.3
WORC-lifestyle	0.85	0.89 (0.81-0.93)	8.7	25.2
WORC-emotions	0.89	0.79 (0.66-0.87)	12.9	35.9

## Discussion

This article presents the Dutch version of the WORC. The translation of an HRQL-questionnaire is not a simple operation as it is subject to one overriding requirement–equivalence between source and target version(s), and subject to two constraints–of time and cost [20]. This paper shows the step-by-step creation of a conceptually equivalent of the WORC by following the MAPI methodology. As stated previously, this translation is not a literal translation. The explanation of the questions section at the end of the questionnaire and the ‘Instructions to patients’ at the beginning of the questionnaire are very valuable supplements. In the Instruction section, patients are advised to make their “best guess” if an item does not pertain to them. The “best guess” leaves some space for free interpretation, however as long as this interpretation is the same each time a patient fills out the questionnaire there is no devaluation of the test. This ‘Instructions to patients’ and the ‘section with explanations to the questions’ are essential to the questionnaire and should not be omitted.

Table 1 shows the differences between the Dutch WORC and the original questionnaire. We believe these differences were due to linguistic or cultural differences, except for question 16 (How much difficulty have you experienced with styling your hair because of your shoulder?). Strictly speaking bald people have no difficulty styling their hair. On the other hand in the instruction section patients were asked to make their “best guess” if an item did not pertain to them. In close consultation with the developer of the test we adjusted the explanation of the question. Question 9 (How much difficulty do you have with someone or something coming in contact with your affected shoulder?), 12 (How much difficulty do you experience working above your head?) and 17 (How much difficulty do you have “roughhousing and horsing around” with family or friends?) have been slightly adjusted, because of linguistic differences. Testing the WORC revealed cultural differences e.g. question 10 (How much difficulty do you experience doing push-ups or other strenuous shoulder exercises because of your shoulder?). In the Netherlands far less elderly perform push-ups or other strenuous shoulder exercises on regular basis. Emphasizing to the patient that they read the instruction section carefully resolved this problem.

Even though the Constant Score has become the most widely used shoulder evaluation instrument in Europe, we are not aware of any validated translation. Validated translations have probably never been performed because it is not purely a patient reported outcome measure since 65% is based on physical examination. A correlation of 0.60 between the objective Constant Score and the subjective Dutch WORC was found. This is in accordance with correlations found in other articles [10,22]. There was a strong reversed correlation demonstrated between experienced shoulder hindrance and the WORC (r = -0.75). There were no floor or ceiling effects present. This indicates that patients with the lowest or highest possible score could be distinguished from each other. Furthermore, the responsiveness of questionnaires with substantial floor or ceiling effects would be limited, because changes over time cannot be measured in these patients [35].

### Agreement and reliability

The Crohnbach’s Alpha and intraclass correlation coefficients for this Dutch version were high (ICC 0.91; α 0.95) showing excellent internal consistency and strong test-retest reliability. Results are comparable to the original questionnaire (ICC 0.96) [10] and translations in other countries like Persia (ICC 0.90; α 0.92) [25], Turkey (ICC 0.98; α 0.92) [22], Germany (α 0.96) [23], Norway (ICC 0.84; α 0.91) [21] and Brazil (ICC 0.97; α 0.88) [24]. SEM and SDC in this study are acceptable. The larger the SEM the lower the precision of the instrument. Small changes cannot be distinguished from measurement error when the measurement error is large [40].

The authors of the original WORC suggested that the WORC would be an appropriate measurement tool for primary outcome in clinical trials evaluating treatments in this patient population. Additionally it could be used in clinical practice for following individual patients [10]. Holtby et al. stated that the WORC could provide valuable information to examiners in detecting which patients might not respond favorably to treatment or might require a different management [41]. In the current study the WORC is validated in a heterogeneous population with DRC (rotator cuff tear, calcific tendonitis, tendonitis and impingement). We expect that further validation of the WORC in more specific patient groups and responsiveness of the WORC will give more information about the usage of it in individual patients.

## Conclusion

This study presents the cross-cultural Dutch equivalent of the WORC and elaborates its realization process. The Dutch version of the WORC seems to be a reliable measurement tool for assessing health-related quality of life in patients with rotator cuff disorders within the Dutch population.

## Competing interests

The authors declare that they have no competing interests. No benefits or funds were received in support of this study.

## Authors’ contributions

RW: Designed the study; Member of the translating team; Data analysis and interpretation; Preparation of the manuscript. NW: Preparation of the manuscript; Data analysis and interpretation; Reviewing and editing manuscript. AF: Preparation of the manuscript; Data analysis and interpretation; Reviewing and editing manuscript. HM: Designed the study; Data interpretation; Reviewing and editing manuscript. HS: Member of the translating team; Reviewing and editing manuscript. SG: Member of the translating team; Data interpretation; Reviewing and editing manuscript. RB: Designed the study; Data interpretation; Reviewing and editing manuscript. All authors read and approved the final manuscript.

## Authors’ information

SG is one of the authors and the copyright holder of the original WORC questionnaire.

## Pre-publication history

The pre-publication history for this paper can be accessed here:

http://www.biomedcentral.com/1471-2474/14/362/prepub

## Supplementary Material

Additional file 1 Dutch WORC. Click here for file
