# Supplementary material for: The conceptually equivalent Dutch version of the Western Ontario Rotator Cuff Index (WORC)©
Source: BMC Musculoskelet Disord. 2013 Dec 21;14:362. doi: 10.1186/1471-2474-14-362 (PMC3880030; doi:10.1186/1471-2474-14-362)
Supplement: Additional file 1 — Dutch WORC. [file 1471-2474-14-362-S1.doc]

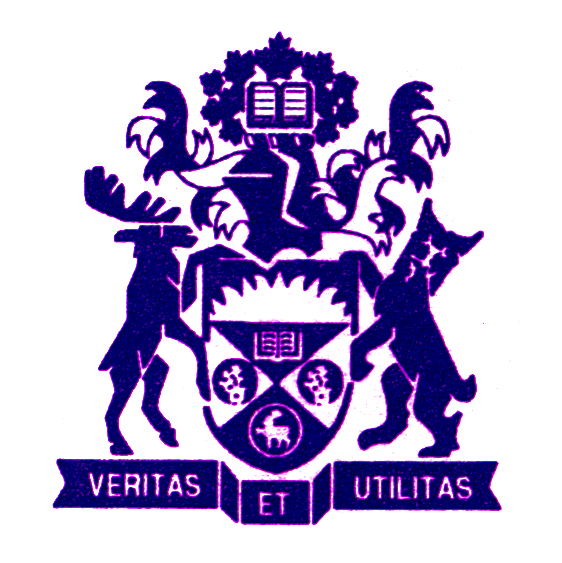


# **WESTERN ONTARIO**

**ROTATOR CUFF INDEX**

**(WORC)©**

**Een ziektespecifiek instrument voor het meten van de kwaliteit van leven**

**bij een aandoening van de rotatorcuff**

Copyright © 1998 (#474673) A. Kirkley MD, S. Griffin, CSS, C. Alvarez, MD

Nederlandse versie: R.N. Wessel, St. Antonius Ziekenhuis ([r.wessel@antoniusziekenhuis.nl](mailto:r.wessel@antoniusziekenhuis.nl))

S. Wiertsema, VU medisch centrum (s.wiertsema@vumc.nl)

Toestemming tot reproductie van de WORC wordt in de regel door de auteurs verleend aan individuele personen en organisaties voor hun eigen gebruik. Een verzoek tot toestemming tot reproductie van de WORC dient te worden gericht aan Sharon Griffin, Coördinator, Fowler Kennedy Sport Medicine Clinic, 3M Centre, University of Western Ontario, London, Ontario Canada N6A 3K7.

Alle rechten voorbehouden. Geen deel van dit meetinstrument mag in welke vorm of door wat voor methode dan ook worden gereproduceerd of overgedragen - elektronisch, mechanisch, met inbegrip van fotografie, opname, of opslag in een informatiesysteem of geautomatiseerde gegevensbank - zonder toestemming van de houder van het copyright. Hierbij wordt de houder van dit instrument toestemming verleend tot reproductie van het WORC-scoringsalgoritme voor eigen gebruik.

Literatuur: Kirkley A, Alvarez C, Griffin S. The Development and Evaluation of a Disease-Specific Quality of Life Measurement Tool for Rotator Cuff Disease: The Western Ontario Rotator Cuff Index, Clinical Journal of Sport Medicine 13(2):84-92,2003.

Wiertsema SH, Rietberg MB, Hekman KM, Schothorst M, Steultjens MP, Dekker J. Reproducibility of the Dutch version of the Western Ontario rotator cuff Index, J Shoulder Elbow Surg. 2012 Jun 27 [Epub ahead of print]

Wessel RN, Wolterbeek N, Fermont A, van Mameren H, Sonneveld H, Griffin S, de Bie RA. Equivalent Dutch version of the Western Ontario Rotator Cuff Index (WORC)©.

**INSTRUCTIES VOOR DE PATIËNT**

In de volgende vragenlijst wordt u gevraagd om vragen te beantwoorden en uw antwoord te geven door **één** schuine streep (/) op de horizontale lijn te zetten,

zoals hieronder aangegeven.

**OPMERKING:**

1. Als u de schuine streep (/) helemaal links op de lijn zet,

dan geeft u daarmee aan dat u geen pijn heeft.

2. Als u de schuine streep (/) helemaal rechts op de lijn zet,

dan geeft u daarmee aan dat u extreem veel pijn heeft.



3. Let wel:

a) hoe verder u de schuine streep naar rechts zet, hoe **meer** last u van het

genoemde symptoom heeft.

b) hoe verder u de schuine streep naar links zet, hoe **minder** last u

van het genoemde symptoom heeft.

**c) zet uw schuine streep ( / ) niet buiten de gemarkeerde uiteinden**

U wordt gevraagd om op deze vragenlijst aan te geven hoeveel last u in de afgelopen week van een bepaald symptoom heeft gehad in relatie tot uw aangedane schouder. Als u er niet zeker van bent welke schouder is aangedaan of als u andere vragen heeft, zorg dan dat alles duidelijk is voordat u de vragenlijst gaat invullen.

Als u om wat voor reden dan ook een vraag niet begrijpt of naar uw idee voor meerdere uitleg vatbaar is, raadpleeg dan de uitleg die u aan het einde van de vragenlijst aantreft. Vervolgens kunt u uw schuine streep ( / ) op de juiste plaats

op de horizontale lijn zetten. **Als een vraag niet op u van toepassing is of als u de afgelopen week geen last heeft gehad van de genoemde klacht, geef dan een antwoord dat voor uw gevoel het dichtst bij de waarheid komt. Het is belangrijk dat u alle vragen beantwoordt.**

**Deel A: Lichamelijke klachten**

**AANWIJZINGEN VOOR PATIËNTEN**

**De volgende vragen gaan over de lichamelijke klachten die u als gevolg van uw schouderprobleem ondervindt. Wilt u bij de onderstaande vragen aangeven in hoeverre u de lichamelijke klachten heeft ervaren gedurende de afgelopen week? (Beantwoord de vragen door één** schuine streep (/) **op de lijn te zetten)**

1. Hoeveel stekende pijn ervaart u in uw schouder?

geen extreem

pijn veel pijn

1. Hoeveel constante, zeurende pijn ervaart u in uw schouder?

geen extreem

pijn veel pijn

1. Hoeveel krachtverlies ervaart u in uw schouder?

geen extreem veel

krachtverlies krachtverlies

1. Hoeveel stijfheid of bewegingsbeperking ervaart u in uw schouder?

geen extreem veel

1. Hoeveel klikken, kraken of knarsen ervaart u in uw schouder?

geen extreem

veel

1. Hoeveel last ervaart u in de spieren van uw nek vanwege uw

aangedane schouder?

geen extreem

last veel last

**DEEL B: Sport/Recreatie**

**AANWIJZINGEN VOOR PATIËNTEN**

**De volgende vragen gaan over de invloed die uw schouderprobleem heeft gehad op het uitoefenen van uw sportieve en recreatieve activiteiten gedurende de afgelopen week.** **(Beantwoord de vragen door één** schuine streep (/) **op de lijn te zetten)**

1. Hoeveel invloed heeft uw schouderprobleem op uw lichamelijke conditie?

geen extreem veel

invloed invloed

1. Hoeveel invloed heeft uw schouderprobleem op de mogelijkheid om hard of ver te

gooien?

geen extreem veel

invloed invloed

1. Hoe bang bent u dat iets of iemand tegen uw geblesseerde schouder stoot?

niet extreem

bang bang

1. Hoeveel last heeft u met opdrukken of andere zware schouderoefeningen vanwege

uw schouderprobleem?

geen extreem

last veel last

**DEEL C: WERK**

**AANWIJZINGEN VOOR PATIËNTEN**

**De volgende vragen gaan over de invloed die uw schouderprobleem heeft gehad op het uitoefenen van uw werkzaamheden binnen- en buitenshuis gedurende de afgelopen week.** **(Beantwoord de vragen door één** schuine streep (/) **op de lijn te zetten)**

1. Hoeveel last ervaart u met uw dagelijkse activiteiten in of om het huis of tuin?

geen extreem

last veel last

1. Hoeveel last ervaart u bij het verrichten van werkzaamheden boven

schouderhoogte?

geen extreem

last veel last

1. Hoe vaak gebruikt u uw andere arm om uw geblesseerde arm te compenseren?

helemaal constant

niet

1. Hoeveel last ervaart u bij het tillen van zware voorwerpen op of onder schouderhoogte?

geen extreem

last veel last

**DEEL D: Manier van leven**

**AANWIJZINGEN VOOR PATIËNTEN**

**De volgende vragen gaan over de mate waarin uw schouderprobleem uw manier van leven heeft beïnvloed of veranderd. Geef opnieuw** met één schuine streep ( / ) **aan in hoeverre er in de afgelopen week sprake was van onderstaande problemen.**

1. Hoeveel last heeft u bij het slapen vanwege uw schouder?

geen extreem

last veel last

1. Hoeveel last heeft u bij het verzorgen van uw haar vanwege uw schouder?

geen extreem

last veel last

1. Hoeveel last heeft u met ‘stoeien en ravotten’ met familie/vrienden vanwege uw

schouder?

geen extreem

last veel last

1. Hoeveel last heeft u bij het aan- en uitkleden vanwege uw schouder?

geen extreem

last veel last

**DEEL E: Emoties**

**AANWIJZINGEN VOOR PATIËNTEN**

**De volgende vragen hebben betrekking op uw gemoedstoestand gedurende de afgelopen week vanwege uw schouderprobleem. Beantwoord de vragen door één** schuine streep ( / ) **te zetten.**

1. Hoeveel frustratie voelt u vanwege uw schouder?

geen extreem veel

frustratie frustratie

1. In hoeverre voelt u zich depressief of ‘in de put zitten’ vanwege uw schouder?

niet extreem

1. Hoeveel zorgen maakt u zich over het effect van uw schouderprobleem op uw

werk?

geen extreem veel

zorgen zorgen

**HARTELIJK DANK VOOR HET INVULLEN VAN DE VRAGENLIJST**

**Uitleg van de betekenis van de vragen in de Western Ontario Rotator Cuff (WORC) Index**

Deel A: Lichamelijke symptomen

Vraag 1:

Heeft betrekking op snel en plotseling opkomende pijn in uw schouder of pijn die u als scherp/stekend zou omschrijven.

Vraag 2:

Heeft betrekking op doffe achtergrondpijn die er altijd lijkt te zijn in tegenstelling tot scherpe pijn zoals bedoeld bij vraag 1.

Vraag 3:

Heeft betrekking op een gebrek aan kracht om een beweging uit te voeren.

Vraag 4:

Heeft betrekking op het gevoel dat het gewricht niet wil bewegen. Dit wordt vaak ’s morgens bij het opstaan, na lichamelijke inspanning of na een periode van inactiviteit ervaren. Het zou ook kunnen verwijzen naar het niet kunnen beschikken over het gehele bewegingsbereik van uw schouder in alle of een bepaalde richting(en).

Vraag 5:

Heeft betrekking op één van deze geluiden of iets wat u in uw schouder voelt bij een willekeurige beweging.

Vraag 6:

Heeft betrekking op de mate van spanning, pijn, spasme die u voelt in de spieren van uw nek en die lijkt te worden veroorzaakt door uw schouderprobleem.

Deel B: Sport/Recreatie

Vraag 7:

Heeft betrekking op het conditiepeil dat u had voordat u last kreeg van uw schouder. Omvat een afname van de spierspanning of -kracht, lichamelijke conditie of kracht.

Vraag 8:

Heeft betrekking op een activiteit boven uw macht, waarvan u voor de uitvoering enige kracht nodig heeft. Als u nooit een bal gooit, denk dan aan andere activiteiten waarbij u een werpbeweging moet maken, zoals smashen bij het volleyballen, een stok naar uw hond gooien, borstcrawlen, serveren bij het tennissen, etc.

Vraag 9:

Ga bij uzelf na of u al eens bang of bezorgd bent geweest dat iemand of iets u raakt of in aanraking komt met uw aangetaste schouder, zoals tijdens het sporten, in een kamer vol mensen, in een lift of iemand die u ter begroeting op de schouder slaat.

Vraag 10:

Heeft betrekking op lichamelijke inspanning waarbij u uw schouder moet belasten, zoals opdrukken, bankdrukken, etc.

Deel C: Werk

Vraag 11:

Dit heeft betrekking op activiteiten als harken, scheppen, stofzuigen, afstoffen, wieden, schoffelen en de ramen of vloeren wassen etc.

**Verklaring van vragen, vervolg**

Vraag 12:

Heeft betrekking op activiteiten waarbij u uw armen boven uw schouders moet tillen, zoals bij het inruimen van borden in een kast, reiken naar een voorwerp, het plafond schilderen of schilderen boven schouderniveau, etc.

Vraag 13:

Heeft betrekking op de vraag of u nu uw andere arm gebruikt voor een bepaalde activiteit of werkzaamheden waar u anders de arm voor zou gebruiken aan de aangedane kant. Als uw andere schouder ook symptomen vertoont van een aandoening aan de ‘rotator cuff’ of een andere aandoening, overweeg dan hoe u de vraag zou beantwoorden als die schouder gezond was.

Vraag 14:

Dit heeft geen betrekking op het optillen van voorwerpen boven uw hoofd, maar op het optillen van zware voorwerpen onder schouderhoogte, zoals het tillen van een tas met boodschappen, een krat met frisdrank, koffer, apparatuur op het werk, boeken, etc.

Deel D: Manier van leven

Vraag 15:

Heeft betrekking op het moeten veranderen van uw slaaphouding, het 's nachts wakker worden, moeite hebben om in slaap te komen of niet-uitgerust wakker worden.

Vraag 16:

Heeft betrekking op alles wat er bij haarverzorging komt kijken, zoals kammen, borstelen of wassen, waarbij u uw aangedane arm moet optillen. Indien u schouderklachten heeft, maar daar geen last van heeft bij de verzorging van uw haar (bijvoorbeeld omdat dat prima gaat met uw andere arm), zet u een streepje links op de lijn. Als u geen problemen ervaart met de verzorging van uw haar omdat u kalend bent, geef dan aan hoeveel problemen u ervaart bij het wassen van de bovenkant van uw hoofd.

Vraag 17:

Heeft betrekking op elke activiteit met familie (bijvoorbeeld kleinkinderen) of vrienden waarbij enige mate van ruw en speels lichaamscontact aan de orde is.

Vraag 18:

Heeft betrekking op bewegingen naar de rug om bijvoorbeeld een rits of een knoop open of dicht te doen, een beha aan of uit te doen, een trui(tje) over uw hoofd aan of uit te trekken of een shirt of truitje in uw broek te stoppen.

Deel E: Gevoelens

Vraag 19:

Heeft betrekking op gevoelens van frustratie omdat u dingen niet kunt doen die u voorheen altijd wel deed of die u wilt, maar niet kunt doen.

Vraag 20:

In de put zitten of zich depressief voelen, speken voor zich.

Vraag 21:

Heeft betrekking op de mate waarin u zich zorgen maakt dat uw schouderprobleem zal verergeren in plaats van verbeteren of stabiel zal blijven en het effect dat dat zal hebben op de uitoefening van uw beroep of werkzaamheden (houd daarbij rekening met werkzaamheden binnen en buitenshuis).

SCORE BEPALEN VAN DE WESTERN ONTARIO ROTATOR CUFF (WORC) INDEX

1. Meet de afstand vanaf de linkerzijde van de streep en bereken de score uit een maximum van 100 (tot de halve mm nauwkeurig). Noteer het resultaat in de daartoe voorziene ruimte voor die vraag.
2. U kunt een totaalscore berekenen voor elk afzonderlijk deelgebied (Lichamelijke symptomen/600; Sport en recreatie/400; Werk/400; Levensstijl/400; Emoties/300) of de scores van de afzonderlijke deelgebieden optellen voor een totaalscore van maximaal 2100.
3. Sommigen hechten meer belang aan een score uit 100, met andere woorden de score uitgedrukt als een percentage. Aangezien de slechtst mogelijke score 2100 is, wordt de totaalscore afgetrokken van 2100 en gedeeld door 2100 x 100 om het percentage te krijgen. Als uw patiënt bijvoorbeeld een opgetelde totaalscore heeft van 1625, dan is de percentagescore:

$$\frac{2100 - 1625}{2100}\times100=22.6\%$$

Hetzelfde geldt voor elk afzonderlijk deelgebied.

Lichamelijke symptomen Sport/recreatie Werk Levensstijl

L 15 ________.____

L 16 ________.____

L 17 ________.____

L 18 ________.____

TOTAAL

_______.____

LS 1 ________.____

LS 2 ________.____

LS 3 ________.____

LS 4 ________.____

LS 5 ________.____

LS 6 ________.____

TOTAAL

__________._____

S 7 ________.____

S 8 ________.____

S 9 ________.____

S 10 ________.____

TOTAAL

__________.____

W11________.____

W12________.____

W13________.____

W14 ________.____

TOTAAL

_________._____

Emoties Totaal

LS ________.____

S ________.____

W ________.____

L ________.____

G ________.____

TOTAAL:

__________._____

G 19 ________.____

G 20 ________.____

G 21 ________.____

TOTAAL _______.____
